# Impact of CO_2_ leakage from sub-seabed carbon dioxide capture and storage (CCS) reservoirs on benthic virus–prokaryote interactions and functions

**DOI:** 10.3389/fmicb.2015.00935

**Published:** 2015-09-07

**Authors:** Eugenio Rastelli, Cinzia Corinaldesi, Antonio Dell’Anno, Teresa Amaro, Ana M. Queirós, Stephen Widdicombe, Roberto Danovaro

**Affiliations:** ^1^Department of Environmental and Life Sciences, Polytechnic University of Marche, AnconaItaly; ^2^Stazione Zoologica Anton Dohrn, NaplesItaly; ^3^Hellenic Center for Marine Research, HeraklionGreece; ^4^Norwegian Institute for Water Research, BergenNorway; ^5^Plymouth Marine Laboratory, PlymouthUK

**Keywords:** viral infection, benthic prokaryotes, biogeochemical cycles, climate change, heterotrophic carbon production, enzymatic activity

## Abstract

Atmospheric CO_2_ emissions are a global concern due to their predicted impact on biodiversity, ecosystems functioning, and human life. Among the proposed mitigation strategies, CO_2_ capture and storage, primarily the injection of CO_2_ into marine deep geological formations has been suggested as a technically practical option for reducing emissions. However, concerns have been raised that possible leakage from such storage sites, and the associated elevated levels of pCO_2_ could locally impact the biodiversity and biogeochemical processes in the sediments above these reservoirs. Whilst a number of impact assessment studies have been conducted, no information is available on the specific responses of viruses and virus–host interactions. In the present study, we tested the impact of a simulated CO_2_ leakage on the benthic microbial assemblages, with specific focus on microbial activity and virus-induced prokaryotic mortality (VIPM). We found that exposure to levels of CO_2_ in the overlying seawater from 1,000 to 20,000 ppm for a period up to 140 days, resulted in a marked decrease in heterotrophic carbon production and organic matter degradation rates in the sediments, associated with lower rates of VIPM, and a progressive accumulation of sedimentary organic matter with increasing CO_2_ concentrations. These results suggest that the increase in seawater pCO_2_ levels that may result from CO_2_ leakage, can severely reduce the rates of microbial-mediated recycling of the sedimentary organic matter and viral infections, with major consequences on C cycling and nutrient regeneration, and hence on the functioning of benthic ecosystems.

## Introduction

Atmospheric CO_2_ emissions are driving ocean warming and acidification with consequences for marine biodiversity and ecosystem functioning. This is causing increasing concern among researchers, policy makers and the public alike, especially in light of the ongoing increase in atmospheric CO_2_ concentrations expected under the majority of future emission scenarios (Orr et al., 2005; IPCC, 2014).

Carbon dioxide (CO_2_) capture and storage (CCS) has been proposed as a technical method to reduce CO_2_ emissions and thus mitigate the growing climate impacts, by concentrating and injecting the CO_2_ into specifically dedicated geological reservoirs (Schrag, 2009). Due to issues of accessibility, the majority of these reservoirs are sub-seafloor geological formations in coastal areas, raising concerns over the possibility that such activities could lead to CO_2_ leakage, especially during the period of active injection (Shaffer, 2010; de Vries et al., 2013; Taylor et al., 2014). The effects of CO_2_ leaking from sub-seafloor reservoirs on the marine environment includes localized seawater and sediment acidification as well as a number of changes in other physical–chemical properties (Gehlen et al., 2011; Lichtschlag et al., 2014; Queirós et al., 2014; Rodríguez-Romero et al., 2014), many of which have been shown to alter benthic assemblages and biogeochemical processes (Laverock et al., 2013; Widdicombe et al., 2013, 2015; Tait et al., 2014). These studies have also highlighted a variety of responses amongst different species and taxa, depending on the local hydrodynamic regime, and the duration/extent of the leakage (de Vries et al., 2013; Widdicombe et al., 2013; Blackford et al., 2014). Most studies reported also that the scale of the impact on benthic biota increases with increasing CO_2_ leakage (Dewar et al., 2013; Phelps et al., 2015; Shitashima et al., 2015), but results are not consistent amongst different storage sites and with changing seasons (Jones et al., 2015). The large variability in the biological response to CO_2_ is particularly evident in coastal ecosystems where, compared to the deep sea, the lower pressures, higher temperatures and the high variability of the physical–chemical variables can alter the leakage process and the biotic components. A number of uncertainties still surround efforts to predict the impact of leakage from CCS, and this limits our ability to evaluate costs vs. benefits and to make final decisions about the suitability of the CCS approach (de Vries et al., 2013).

The effects of CO_2_ leakage on benthic microbial processes has received limited attention so far (Jones et al., 2015). Results from short-term *in situ* experiments (benthic chambers) of the impact of elevated CO_2_ revealed that high CO_2_ concentrations increased the relative importance of Archaea over Bacteria (Ishida et al., 2013). Whilst two separate sets of chamber experiments conducted on water samples provided contrasting results in terms of the impact of elevated CO_2_ on bacterial C production (Coffin et al., 2004; Piontek et al., 2013). However, while available information indicates that elevated levels of CO_2_ can impact upon microbial organisms and processes, no data are currently present to determine the likelihood of impacts on virus–host interactions under such CO_2_-leakage scenarios. Marine viruses play a key role in biogeochemical processes (Suttle, 2007; Danovaro et al., 2008; Jover et al., 2014; Dell’Anno et al., 2015) and sediments are hot-spots of viral infections with rates up to 1,000 times higher than in the overlying water column (Danovaro and Serresi, 2000; Danovaro et al., 2008). Since all biotic components are potentially affected by viruses, the effect of any environmental stressor (such as CO_2_) on marine viruses could have important consequences far beyond this specific component of the ecosystem.

In the present study, we used a controlled mesocosm experiment to investigate the impact of a simulated CO_2_ leakage on benthic microbial assemblages, with focus, for the first time, on virus–host interactions and their implications on organic matter cycling.

## Materials and Methods

### High-CO_2_ Experimental Setup and Sample Processing

Acidification experiments were conducted over a period of 140 days to simulate the potential effects on benthic ecosystems of the spreading above marine sediments of CO_2_-enriched seawater plumes originating from a CCS reservoir or pipeline. Sediment samples were collected during August 2012 in the Oslofjord coastal area (59°49.4788′ N, 10°58.8595E), Norway, at 100-m depth, using a KC Denmark box corer. We retrieved a total of 50 independently collected liners (i.e., cores with 0.1 m × 0.1 m surface area each and average sediment penetration of ~40 cm), which were then immediately transferred to the benthic mesocosm system at the Norwegian Institute of Water Research, Solbergstrand, Norway. The experimental mesocosms were setup according to Widdicombe et al. (2009). Briefly, all liners were kept in complete immersion during transport to the mesocosm system to prevent desiccation and temperature changes, and then placed in a flow-through holding basin filled with seawater to a depth of 1 m for ~4 weeks for acclimatization. A pipeline situated at 60 m in the adjacent fjord continuously supplied the holding basin with filtered natural seawater. The liners were then distributed randomly and in equal numbers amongst CO_2_ treatments, replicating increasing levels of acute acidification in relation to the natural (control) conditions at the collection site. Five concentrations were used to mimic the impact of plumes of acidified seawater emanating from a theoretical point source leak of CO_2_ from a sub-seabed geological reservoir. The levels chosen were 400 ppm (control) and 1,000, 2,000, 5,000, and 20,000 ppm, according to a hypothesized pCO_2_ decreasing gradient from the site of leakage (20,000 ppm) to the unaffected area (400 ppm). Each liner received a constant supply of seawater within the mesocosm at a flow rate of 120 mL min^-1^. The day light regime in the basin was 8:16 h (light–dark). Sampling took place at the beginning of the experiments (0 days), after 14 days and again after 140 days to assess the short-term long-term effects of the CO_2_ exposure. Seawater acidification was achieved as described by Widdicombe et al. (2009). Briefly, CO_2_ gas (supplied in the form of very fine bubbles, enabling their rapid solution in the seawater) passed through large (450 L) reservoir tanks filled with natural seawater. The CO_2_ flux was regulated via an automated feedback relay system (Walchem) to maintain a set pH level, and the reservoir tanks were continuously supplied with natural seawater (pH~8.1). The required pH levels for each desired pCO_2_ treatment were calculated using CO2Sys based on the alkalinity values measured from the supply seawater. Seawater temperature, salinity, oxygen concentration and pH were monitored three times a week in each liner and in header tanks, using macro probes. The pH in the sediments was acquired in two replicate liners per treatment using Dual Lifetime Referencing based optical sensors, with sampling and calibration and temperature compensation methods described in Queirós et al. (2014).

Sediment samples from the top 0-1-cm sediment layer were collected by using sterile Plexiglas^®^ tubes (Polymethyl methacrylate; 5.5 cm inside diameter) to investigate benthic prokaryotic variables, virus–host interactions and sedimentary organic matter content, composition, and degradation rates. The sediment samples were immediately processed for the analysis of viral and prokaryotic abundance, and for the determination of phytopigments, lipids, carbohydrates, and proteins concentration as detailed below. The analyses of viral production, prokaryotic heterotrophic carbon production and extracellular enzymatic activity (aminopeptidase) were conducted by means of time course experiments at *in situ* temperature as following described. All variables were analyzed in at least three replicates, and all data were normalized to sediment dry weight after desiccation (48 h at 60°C).

#### Phytopigments, Organic Matter Composition, and Extracellular Enzymatic Activities

Phytopigments were extracted in 5 ml of 90% acetone (12 h at 4°C in the dark) and analyzed fluorometrically for the determination of chlorophyll-*a* concentrations. Samples were then added with 200 μl of 0.1 N HCl and analyzed fluorometrically for the determination of phaeopigments concentrations.

The concentration of proteins, carbohydrates, and lipids was measured spectrophotometrically in the top 1-cm sediment layer (Danovaro, 2010). The sum of the carbohydrate, protein, and lipid concentrations converted into carbon equivalents (by using the conversion factors of 0.40, 0.49, and 0.75 μg C μg^-1^, respectively) was defined as biopolymeric carbon (BPC; Pusceddu et al., 2009; Danovaro, 2010).

The extracellular aminopeptidase activities (used as proxy of potential mobilization and consequent utilization of proteins) were determined by the analysis of the cleavage rates of the artificial fluorogenic substrate L-leucine-4-methylcoumarinyl-7-amide (Leu-MCA, Sigma Chemicals) under saturating substrate concentrations (Danovaro, 2010). Briefly, sediment sub-samples were diluted with 0.02-μm pre-filtered seawater collected at the water-sediment interface from each liner, and incubated in the dark at the *in situ* temperature for 1 h. The fluorescence of the samples was measured fluorometrically (380 nm excitation, 440 nm emission), immediately after the addition of the substrate and after the incubation, and converted into enzymatic activity using standard curves of 7-amino-4-methylcoumarin (Sigma Chemicals). The amount of the artificial fluorogenic substrate hydrolyzed by proteases were converted into protein degradation rates using 72 μg of C per micromole of substrate hydrolyzed.

The cell-specific degradation rates were calculated by the ratio of aminopeptidase activity and the corresponding total prokaryotic abundance in each sample.

The turnover times of proteins in the sediment, used as a proxy of protein cycling efficiency, were calculated as the ratio of the whole protein concentrations and their degradation rates converted into C equivalents.

#### Total Prokaryotic Abundance

Total prokaryotic abundance was determined by epifluorescence microscopy as described in Danovaro et al. (2001). Briefly, the sediment samples were treated by ultrasounds (Branson Sonifier 2200, 60W) three times for 1 min after addition of 0.2 μm pre-filtered tetrasodium pyrophosphate solution (final concentration, 5 mM), then properly diluted before filtration onto 0.2 μm pore-size Nuclepore black filters (Whatman). Filters were then stained with SYBR Green I (Sigma Chemicals) by adding, on each filter, 20 μl of the stock solution (previously diluted 1:20 with 0.2 μm pre-filtered Milli-Q water), washed twice with 3 ml sterilized Milli-Q water and mounted onto microscope slides. Filters were analyzed using epifluorescence microscopy (Zeiss Axioskop 2MOT, magnification 1,000×). For each filter, at least 20 microscope fields were observed and at least 400 cells counted.

#### Viral Abundance, Production, and Virus-Induced Prokaryotic Mortality

Viral abundance was determined by epifluorescence microscopy according to the procedure described in Noble and Fuhrman (1998) and applied to the sediments as described in Danovaro (2010). The sediment samples were sonicated three times (Branson Sonifier 2200, 60W) for 1 min after addition of 0.02 μm pre-filtered tetrasodium pyrophosphate solution in seawater (final, 5 mM). In order to eliminate uncertainties in virus counting due to extracellular DNA interference, sub-samples were supplemented with DNase I from bovine pancreas (10 U mL^-1^ final concentration) and incubated for 15 min at room temperature. The samples were properly diluted with 0.02-μm pre-filtered seawater, filtered onto 0.02-μm-pore-size Al_2_O_3_ filters (Anodisc; diameter 25 mm) and then stained with 100 μl of SYBR Gold 2x (diluting stock solution with 0.02-μm pre-filtered TE buffer (10 mM Tris-HCl, 1 mM EDTA). Filters were incubated in the dark for 20 min, rinsed three times with 3 ml of 0.02-μm pre-filtered Milli-Q water, dried under laminar flow hood and then mounted on glass slides with 20 μl of antifade solution (50% phosphate buffer pH 7.8, 50% glycerol, 0.5% ascorbic acid). Viral counts were obtained by epifluorescence microscopy (Zeiss Axioskop 2MOT, magnification 1,000×) examining at least 20 fields per slide, and at least 400 viral particles per filter.

Viral production rates were determined by time-course experiments using the dilution approach (Dell’Anno et al., 2009). Briefly, replicate samples (*n* = 3) for viral counts were collected immediately after dilution of the sediments and after 1–3, 3–6, and 12 h of incubation in the dark at *in situ* temperature. Subsamples were then analyzed as reported for the determination of viral abundance. Virus-induced prokaryotic mortality (VIPM) was calculated as follows:

VIPM = (Viral production/Burst Size) × 100/Prokaryotic Abundance

We assumed a burst size of 45 viruses cell^-1^ reported for marine sediments worldwide (Danovaro et al., 2008).

The C released by the viral shunt was calculated by converting the number of killed prokaryotes into C content using 20 fg C cell^-1^ as conversion factor (Danovaro, 2010).

### Prokaryotic Heterotrophic C Production

The determination of prokaryotic heterotrophic carbon production was carried out using the method of ^3^[H]–leucine incorporation, according to the procedure described in van Duyl and Kop (1994) as modified in Danovaro (2010). Briefly, sediment sampl were added with 0.2-μm pre-filtered seawater, containing [^3^H]-leucine (68 Ci mmol^-1^; final 0.5–1.0 μM), then incubated in the dark and at the *in situ* temperatures. Time-course experiments over 6 h and concentration-dependent incorporation experiments (from 0.05 to 5.0 μM leucine) were also carried out to define the linearity and the saturation level of the [^3^H]-leucine incorporation, respectively. Blanks (*n* = 3) for each sediment sample were added with ethanol immediately before ^3^[H]-leucine addition. After incubation, samples were supplemented with ethanol (80%), centrifuged, washed again two times with ethanol (80%), and the sediment was finally re-suspended in ethanol (80%) and filtered onto polycarbonate filters (0.2 μm pore size; vacuum <100 mm Hg). Subsequently, each filter was washed four times with 2 ml of 5% TCA, then transferred into a Pyrex tube containing 2 ml of NaOH (2 M) and incubated for 2 h at 100°C. After centrifugation at 800×*g*, 1 ml of supernatant fluid was transferred to vials containing the appropriate scintillation liquid. The incorporated radioactivity in the sediment samples was measured with a liquid scintillation counter (PerkinElmer-Packard Tri-Carb 2100 TR).

#### Statistical Analyses

To test for differences in the investigated variables between different treatments and exposure time, a two-way analysis of variance was conducted using distance-based permutational multivariate analyses of variance (PERMANOVA; Anderson, 2005), after checking the homogeneity of variance using the Cochran test. Treatment and time were used as fixed factors and *post hoc* comparison was carried out when significant (*p* < 0.05) differences were encountered. Statistical analyses were performed using the PRIMER v.6.1. program and the PERMANOVA+ add-on.

## Results

Temperature, salinity and oxygen concentrations during the experiments are reported in **Table 1**. The values of all of these variables did not change significantly among treatments, indicating that the primary differences between treatments were associated with the CO_2_ induced changes to the carbonate chemistry system. The injection of CO_2_ at high concentration caused a significant decrease of pH in all treated mesocosms, both in the water column and within the sediment under leakage-like scenarios (**Table 1**).

**Table 1:** Temperature (T), salinity (S), oxygen concentration (O_2_), and pH in the seawater (SW) and sediment (Sed) in the different high-CO_2_ experimental systems (1,000–20,000 ppm) and in the controls (i.e., 400 ppm), reporting mean values and ranges.

Treatment	T (°C)	S (‰)	O_2_ (mg l^-1^)	pH (SW)	pH (sed)
400 ppm	8.89 ± 2.35	33.82 ± 0.52	7.31 ± 0.35	8.05 ± 0.06	7.99 ± 0.15
1,000 ppm	8.75 ± 1.61 (n.s.)	33.82 ± 0.52 (n.s.)	7.34 ± 0.33 (n.s.)	7.68 ± 0.10 (***)	7.69 ± 0.20 (**)
2,000 ppm	8.70 ± 1.65 (n.s.)	33.82 ± 0.52 (n.s.)	7.34 ± 0.32 (n.s.)	7.47 ± 0.11 (***)	7.57 ± 0.20 (**)
5,000 ppm	8.75 ± 1.54 (n.s.)	33.82 ± 0.52 (n.s.)	7.26 ± 0.33 (n.s.)	7.06 ± 0.08 (***)	7.31 ± 0.11 (***)
20,000 ppm	8.79 ± 2.3 (n.s.)	33.81 ± 0.52 (n.s.)	7.23 ± 0.35 (n.s.)	6.58 ± 0.06 (***)	7.10 ± 0.13 (***)

Significant differences between treatments and control are reported in the table with asterisks indicating **p < 0.01 and ***p < 0.001; n.s., not statistically significant.

### Composition of the Sedimentary Organic Matter

**FIGURE 1:**
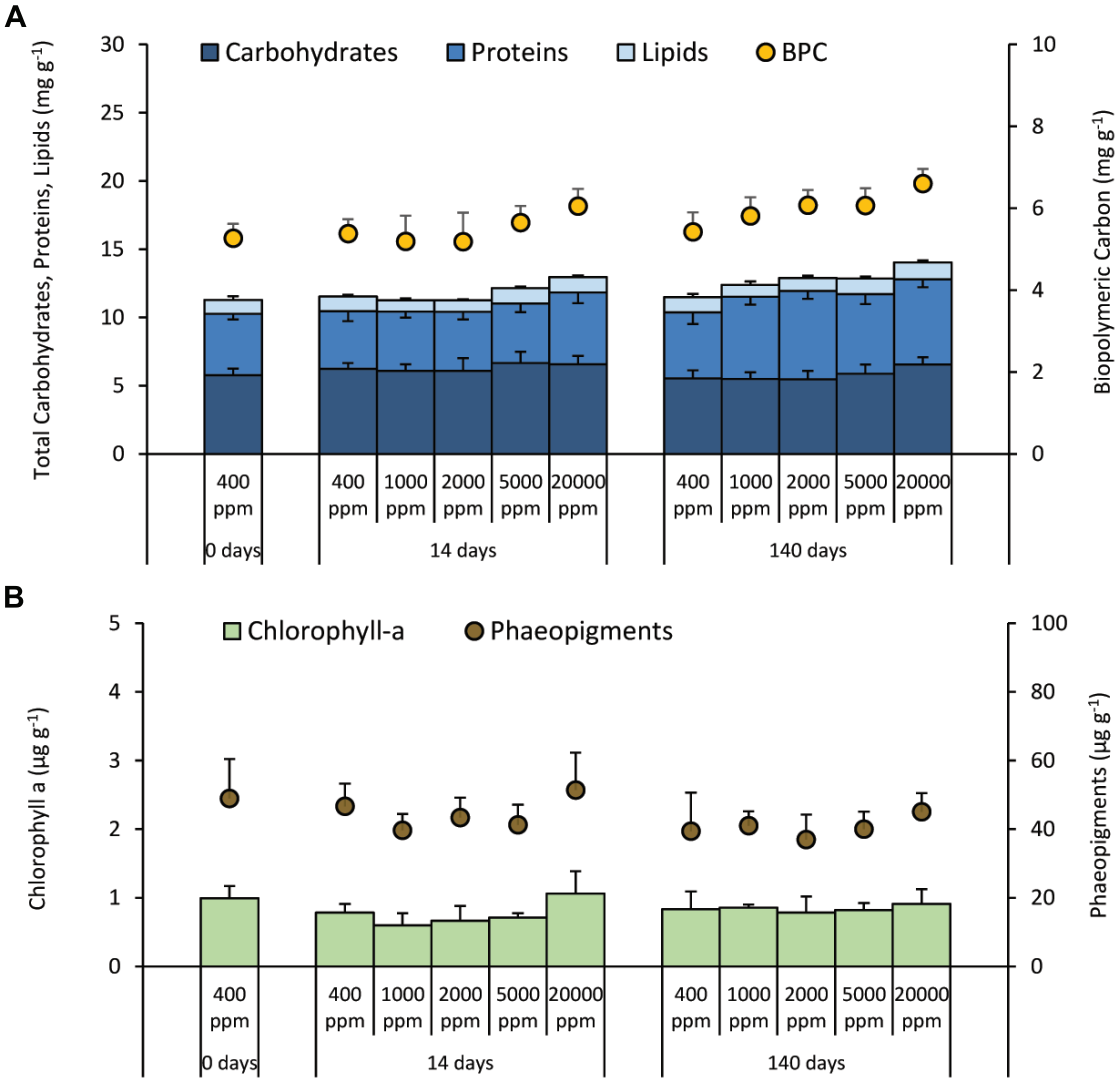
**Quantity and quality of the sediment organic matter.** Reported are the mean values and relative SDs for **(A)** Carbohydrate, protein, and lipid content in surface sediments, as well as the sediment concentration of the Biopolymeric Carbon (BPC), and **(B)** chlorophyll-a and phaeopigment concentration under different high-CO_2_ experimental systems (1,000–20,000 ppm) and in the controls (i.e., 400 ppm).

Carbohydrate and lipid concentrations did not show significant changes either with changing CO_2_ levels or increasing time of exposure (**Figure 1A**). Conversely, protein concentrations showed a progressive increase with increasing time of CO_2_ exposure and, after 140 days, protein concentrations in all acidified sediments were significantly higher than in the control. Biopolymeric C content in the sediments during the experiment showed an increase after 140 days from 7 to 22% at 1,000 and 20,000 ppm when compared with the controls (**Figure 1A**). The concentration of chlorophyll-*a* and phaeopigments did not show significant changes either with changing CO_2_ supply or increasing time of exposure (**Figure 1B**).

### Extracellular Enzymatic Activity

**FIGURE 2:**
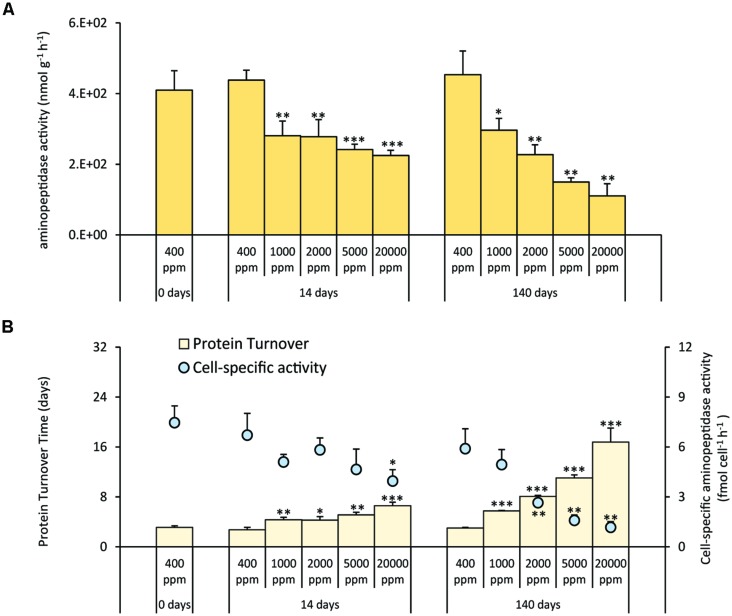
**(A)** Extracellular aminopeptidase activities, and **(B)** cell-specific aminopeptidase activities and sedimentary protein turnover time in the different high-CO_2_ experimental systems (1,000–20,000 ppm) and in the controls (i.e., 400 ppm), showing mean values and relative SDs. Significant differences between treatments and controls are indicated with asterisks: *p < 0.05, **p < 0.01, and ***p < 0.001.

Aminopeptidase activities decreased significantly at all CO_2_ levels after 14 days and were further reduced after 140 days. Such changes were more evident in systems at 5,000 and 20,000 ppm of CO_2_ (**Figure 2A**). The cell-specific activities generally decreased with increasing CO_2_ concentrations and increasing time of exposure. With the exception of CO_2_ treatment at 1,000 ppm, the cell-specific activities in the acidified systems were significantly lower when compared with the controls (**Figure 2B**). As a result, the turnover time of the sedimentary proteins (**Figure 2B**) increased in the acidified systems for up to ca. three times after 14 days of incubation and up to ca. six times after 140 days, with longer turnover times in the most acidified treatment (20,000 ppm).

### Prokaryotic and Viral Abundance and Production

**FIGURE 3:**
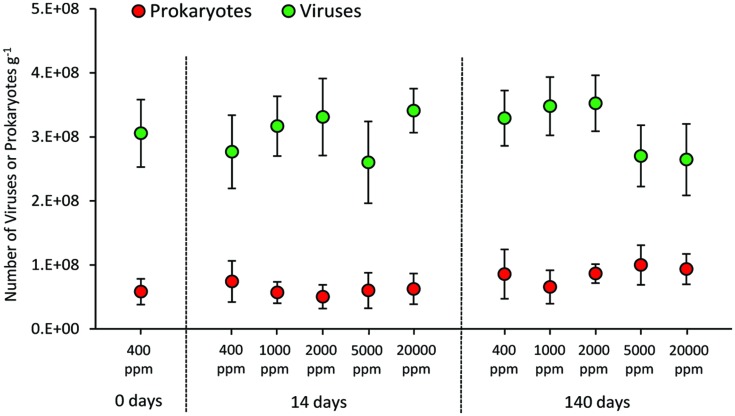
**Total abundance of prokaryotes and viruses in the different high-CO_2_ experimental systems (1,000–20,000 ppm) and in the controls (i.e., 400 ppm), showing mean and SDs values**.

The CO_2_ treatment resulted in no significant changes in the abundance of prokaryotes either in the short and long term (**Figure 3**). Similarly, viral abundance did not change significantly in the short and long term, nor between treated systems (at the different CO_2_ levels) and control (400 ppm).

**FIGURE 4:**
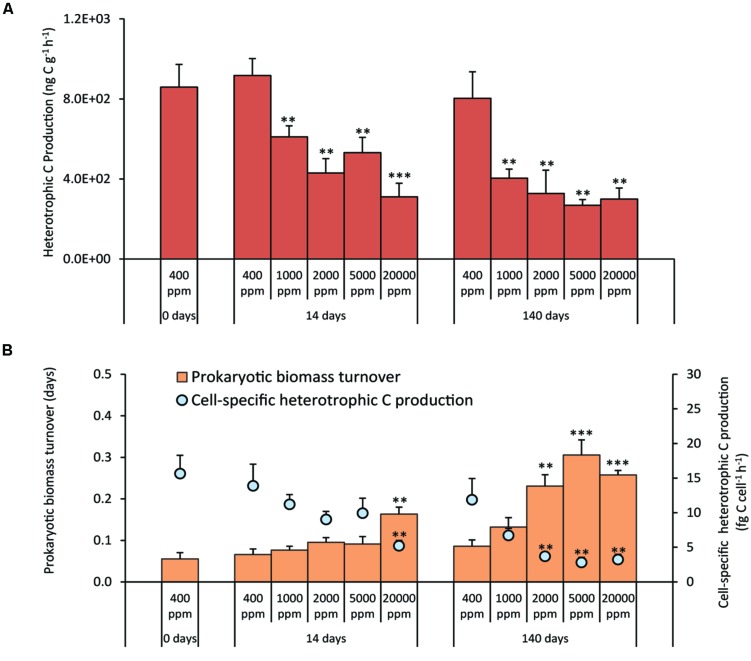
**(A)** Heterotrophic Carbon Production, and **(B)** cell-specific Heterotrophic Carbon Production and prokaryotic biomass turnover time in the different high-CO_2_ experimental systems (1,000–20,000 ppm) and in the controls (i.e., 400 ppm), showing mean values and relative SDs. Significant differences between treatments and controls are indicated with asterisks: **p < 0.01, and ***p < 0.001.

The treatments at different CO_2_ concentrations resulted in a significant decrease of prokaryotic heterotrophic C production either in the short- and long-term experiments, with values 1.5–3.0 times lower than the controls (**Figure 4A**). As a consequence, both in the short and long-term exposure, the cell-specific C production rates decreased (**Figure 4B**). After 140 days, the turnover time of the prokaryotic biomass was significantly longer in the acidified systems than in the controls, with the exception of CO_2_ treatment at 1,000 ppm (**Figure 4B**).

**FIGURE 5:**
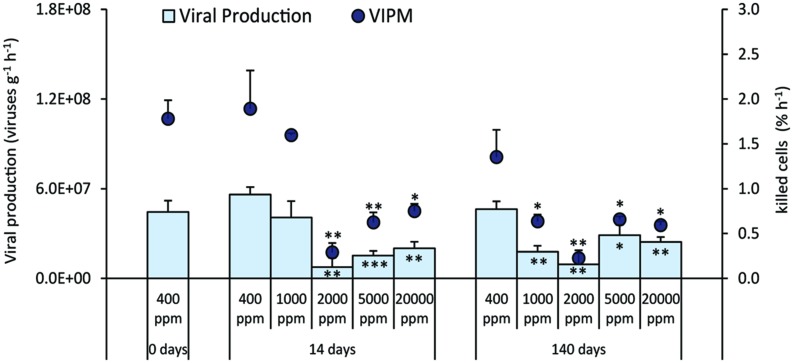
**Rates of viral production and virus-induced prokaryotic mortality (VIPM) in the different high-CO_2_ experimental systems (1,000–20,000 ppm) and in the controls (i.e., 400 ppm), showing mean and relative SDs values.** Significant differences between treatments and controls are indicated with asterisks: *p < 0.05, **p < 0.01, and ***p < 0.001.

Viral production decreased significantly when compared to the control during both the short and long-term experiment, with the exception of CO_2_ treatment at 1,000 ppm after 14 days (**Figure 5**). Similar patterns were observed for the (VIPM), which was significantly lower than in the control (**Figure 5**).

## Discussion

In the present study, we investigated the impact of CO_2_-enriched seawater plumes originated by possible CO_2_ leakages on benthic prokaryotic metabolism and virus–host interactions according to a putative gradient of CO_2_ concentration (from an unaffected area to the site of epicenter leakage).

Previous studies conducted on the impact of CO_2_ leakage on microbial components revealed the presence of significant shifts in the abundance of different microbial components either in the short- or in the long-term exposure (Tait et al., 2014; Watanabe et al., 2014) and using different experimental approaches (Ishida et al., 2013; Blackford et al., 2014).

Conversely, in the present study prokaryotic abundance did not change significantly. Such discrepancies could be related to several factors, including the different experimental set up (e.g., different CO_2_ exposure levels and leakage scenarios), the effects of acidification on the physico-chemical properties of the sediments (e.g., different levels of mobilization of heavy metals or other toxic compounds, potentially impacting the biota; de Orte et al., 2014), or the changing virus–host interactions depending on the ecosystem investigated. We also found that viral abundance, as observed for the abundance of their hosts, was unaffected by CO_2_ leakage.

Despite the fact that we did not observe any significant effect on prokaryotic and viral abundances, we provide evidence that the high-CO_2_ exposure, over a period of 140 days, had a significant impact on the benthic microbial components in functional terms, with no signs of acclimatization to any of the tested CO_2_ leakage scenarios. In our experiments, heterotrophic C production in the sediments, when compared with control values, was significantly reduced at all CO_2_ concentrations. In particular, the prolonged (140 days) exposure at 2,000 ppm determined a decrease of heterotrophic C production to values close to those reported for the treatment at 20,000 ppm. Such an effect was even more evident when heterotrophic C production was normalized to the total prokaryotic abundance, suggesting that CCS-induced CO_2_ leakage reduced significantly the C production efficiency of heterotrophic prokaryotes even at relatively low CO_2_ concentrations (i.e., 2,000 ppm).

The abatement of benthic microbial metabolism was associated with the increase of replication times of prokaryotic cells. In addition, our results on growth rates indicated that prokaryotic turnover was affected not only by the CO_2_ concentrations, but also by the duration of the treatments. Since microbial metabolism plays a crucial role in the functioning of benthic ecosystems, the strong abatement of heterotrophic C production and the slowdown of prokaryotic turnover rates due to CCS-induced CO_2_ leakage, can have important functional implications.

Our experiment provides evidence, for the first time, that viral production and VIPM decreased significantly in the acidified sediments. It is known that there is a strong interconnection between benthic viral replication and host metabolism (Danovaro et al., 2008; Corinaldesi et al., 2012), thus a decrease in prokaryotic metabolism is expected to determine a decrease in viral production, and consequently in prokaryotic mortality rates. We found, indeed, significant relationships between heterotrophic C production and, either viral production (*n* = 33, *r* = 0.860, *p* < 0.01), or VIPM (*n* = 33, *r* = 0.917, *p* < 0.01). A reduced top–down control exerted by viruses could thus explain why prokaryotic abundance remained largely unvaried despite the significant impact of acidification on prokaryotic metabolism.

A further confirmation of the impact of CCS-induced CO_2_ leakage on microbial metabolism was highlighted by the concomitant and progressive decrease of aminopeptidase activities under acidified conditions. This finding was consistent for exposures at all CO_2_ concentrations suggesting that the impact on organic matter degradation rates can be significant even far from the CO_2_ leak epicenter.

Recent findings provide evidence that aminopeptidase activity in the sediment is the main cause of virus decomposition (Dell’Anno et al., 2015). Since viral abundance depends on the balance between the rates of viral production and decay (Corinaldesi et al., 2010), the strong abatement of aminopeptidase activity in acidified sediments can contribute to explain why viral abundance remained rather constant despite the significant decrease of viral replication rates.

We report here a progressive accumulation of proteins (by 21–34%) in the acidified sediments when compared to the controls. The increased concentrations of sedimentary proteins cannot be explained by an increase of the benthic primary production. Indeed, the lack of changes in the concentrations of phytopigments (either chlorophyll-*a* and phaeopigments) among treatments, and comparing treated sediments and their controls, suggests that the high-CO_2_ exposure had no effects on photoautotrophs and the photosynthetic production of organic matter. The increase in sediment protein concentrations in acidified conditions can thus be explained by the decrease of proteases, which indicates the reduced ability of the benthic system to degrade detrital organic matter, as also confirmed by the increase of turnover times of sedimentary protein pools.

Viruses, by killing prokaryotic hosts, convert prokaryotic biomass into organic detritus (through the so-called *viral shunt*, Wilhelm and Suttle, 1999), which is utilized to sustain the metabolism of un-infected prokaryotes (Corinaldesi et al., 2012; Weitz et al., 2015). Our findings indicated that the organic C released by viral shunt in the acidified sediments decreased by ca 2–5 times when compared to the controls. Based on the fact that the viral shunt is known to stimulate the prokaryotic metabolism, turnover rates, and the cycling of the sedimentary organic matter (Danovaro et al., 2008), our results suggest that the concomitant reduction in the VIPM rates can exacerbate such effects, by further lowering prokaryotic metabolism under CO_2_-leakage scenarios. A lower viral “predatory” pressure on prokaryotes indeed could reduce the prokaryotic efficiency in the utilization of organic substrates, and contribute to the effect of accumulation of organic matter in the sediments of acidified systems.

To date the information on the effect of CO_2_-induced acidification on the benthic bacterial and archaeal diversity is extremely scarce (Blackford et al., 2014; Watanabe et al., 2014), and the effects on the diversity of viruses remain completely unknown (Jones et al., 2015). The lack of significant changes in the abundances of prokaryotes and viruses under different levels of CO_2_ exposure in our experiments could indicate high resilience of the original microbial assemblages, or compensating shifts in the benthic microbial diversity with species proliferating, which are favored in acidified conditions. Some authors have hypothesized a possible advantage for archaea (in particular for chemoautotrophs) in acidified sediments (Ishida et al., 2013; Tait et al., 2014). Although the effects of pH shifts on marine viruses are difficult to predict (see Danovaro et al., 2011), possible changes in the relative abundance of specific viral taxa and in the overall diversity of viruses can be certainly expected.

Overall, our data indicate that the impacts of potential leakage form CCS storage sites and pipelines may not be limited to the sediments only in the vicinity of the point-source leak. Indeed, the lateral dispersal of plumes of seawater enriched in CO_2_ over wider areas of marine sediments can significantly reduce the benthic microbial metabolism and organic matter degradation rates, VIPM, and viral shunt, with consequences, thought at local scale, on organic matter cycling and nutrient regeneration.

Our experiment was conducted on sediments collected at ca 100-m depth, but CCS can potentially be located in deep-sea sediments (i.e., at depths > 200 m). Recent findings provided evidence that the viral control over benthic prokaryotic assemblages increases with increasing water depths and that >80% of prokaryotic production is abated by viral infection beneath 1,000-m depth (Danovaro et al., 2008). As a consequence it is possible to hypothesize that the development of CCSs in deep ocean sediments and porous subseabed formations (Ishida et al., 2005; House et al., 2006; Goldberg et al., 2008; Shaffer, 2010; Barry et al., 2013), and the eventual leakages from storage sites, could impact the functioning of deep-sea ecosystems by altering the virus–host interactions and the consequent *equilibria* of the benthic microbial component, with potentially important effects at local scale on biogeochemical processes.

## Conflict of Interest Statement

The authors declare that the research was conducted in the absence of any commercial or financial relationships that could be construed as a potential conflict of interest.
